# Galectin-3 induces vascular smooth muscle cells calcification via AMPK/TXNIP pathway

**DOI:** 10.18632/aging.204130

**Published:** 2022-06-27

**Authors:** Lei Tian, Yong Wang, Ruiyan Zhang

**Affiliations:** 1 Department of Cardiovascular Medicine, Ruijin Hospital, Shanghai Jiao Tong University School of Medicine, Shanghai, China

**Keywords:** galectin-3, TXNIP, calcification, VSMCs

## Abstract

Galectin-3 plays an important role in atherosclerosis. Upregulation of VSMCs calcification is involved in the progression and development of vulnerable plaques. Thioredoxin-interacting protein (TXNIP) has been regarded as an important determinant in regulating inflammation and oxidative stress. In this study, we evaluated the role of TXNIP in galectin-3-induced vascular calcification. A primary culture of mouse VSMCs was established by enzymatic digestion of aorta. Small interfering (si) RNA was used to knock down the expression of target gene. VSMCs were treated with 3-methyladenine (3-MA) or compound C respectively. Western blot was performed to detect the protein level in VSMCs, Alkaline phosphatase (ALP) and Alizarin red staining was used to observe calcium deposition. Dihydroethidium (DHE) staining was used to observe the reactive oxygen species (ROS) production. Here we showed that galectin-3 increased aorta and VSMCs calcification, which was associated with AMPK/TXNIP upregulation and autophagy activation. TXNIP inhibition decreased galectin-3-induced aorta and VSMCs calcification and autophagy activation. 3-MA or Atg5 siRNA decreased galectin-3-induced upregulation of Runx2, BMP2 and OPN. AMPK mediated galectin-3-induced VSMCs osteogenic differentiation. These findings illustrated that TXNIP mediated galectin-3-induced vascular calcification, AMPK and autophagy activation were also associated with this process.

## INTRODUCTION

The incidence of atherosclerosis is increasing over the development countries. Atherosclerotic lesions followed by plaque rupture and myocardial infarction are the primary causes of ACS [1, 2]. Vascular calcification is critical in the development of plaque formation and rupture [3]. Galectin-3, a member of the β-galactoside-binding lectin family, is related to plaque calcification and promotes intimal calcification [4]. In hemodialysis patients, galectin-3 has been proved to be correlated to vascular calcification [5]. In type 2 diabetes mellitus patients, galectin-3 levels were significantly higher in the coronary heart disease (CAD) group than the non-CAD group and were correlated positively with the calcified plaque type [4]. Pugliese et al. also found that galectin-3 promoted vascular calcification, induced sheet-like, lamellated, homogeneous calcium deposition (macrocalcification) and increased plaque stability [6].

TXNIP was originally regarded as a key determinant of cellular sulfhydryl redox homeostasis [7]. Recently, the effects of TXNIP were largely explored, it also participates a variety of biological processes, such as inflammation, oxidative stress, cell apoptosis, and glucose and lipid metabolism [8–10]. The ubiquitously presented thioredoxin (Trx) system is one of the most important antioxidative mechanisms. Trx protects cells against oxidative damage, TXNIP binds and directly interacts with Trx, leading to reactive oxygen species (ROS) increase and oxidative damage in cells [11]. Besides its strong effects in cellular redox, inflammation and energy metabolism, TXNIP also plays an important role in vascular function [7]. In cardiovascular systems, TXNIP upregulation is related to hypertension and arterial stiffness [12, 13]. TXNIP mediates the activation of NLRP3 inflammasome in macrophages [14]. VSMCs is the principal cellular components in the medial layer of arteries. TXNIP inhibition protects VSMCs from cellular oxidative stress, and reduces the interaction between VSMCs and macrophages in the blunted inflammatory response [7]. TXNIP has also been reported to mediate advanced glycation end products- Bovine Serum Albumin (AGEs-BSA)-induced ROS production and VSMC calcification [15].

Autophagy has also been found to play an important role in atherosclerosis. Autophagy accelerates cell growth and survival by passing on metabolic substrates to enable cells to meet their energy requirements [16]. However, in VC process, the role of autophagy is still unclear. Indoxyl sulfate stimulates the autophagy and furtherly induces osteoblast differentiation and matrix mineralization of VSMCs [17]. Low dietary potassium has also been reported to increase elevation of intra-cellular calcium, activate autophagy, and further promote VSMC osteoblast differentiation and calcification [18]. Xu et al. also found that ghrelin increased the expression of LC3 and beclin1, 3-MA delayed hormonal ghrelin induced upregulation of VC amelioration [19].

Until now, the effect of galectin-3 in vascular calcification is still not clear, in particular, we still do not know how TXNIP, an inflammation related protein, regulates vascular calcification. The role of autophagy in VC should also be furtherly determined. The aim of this study was to investigate the role of TXNIP in VSMCs which were treated by galectin-3. The role of autophagy and the activation of related signaling pathways in this process were also explored to determine the possible regulatory mechanisms.

## METHODS

### Reagents

The Dulbecco’s Modified Eagle’s Medium (DMEM), fetal bovine serum (FBS) and penicillin/streptomycin were purchased from Gibco (Carlsbad, CA, USA). Galectin-3 and compound C (AMPK inhibitor) were purchased from Abcam (Cambridge, UK). 3-MA was obtained from Sigma Aldrich (St. Louis, MO, USA). Verapamil was purchased from Hefeng Pharmaceutical (Shanghai, China). The primary antibody against phospho-AMPK, AMPK, phospho-mTOR, mTOR and GAPDH were acquired from the Cell Signaling Technology Inc (Danvers, MA, USA). The primary antibodies against TXNIP, LC3B and Runx2 purchased from Sigma-Aldrich (St. Louis, MO, USA). Anti-galectin-3, anti-P62, anti-NLRP3, anti-caspase-1, anti-IL-1β, anti-OPN and anti-BMP2 were obtained from Abcam. All other chemicals were from commercial sources.

### Cell culture

A primary culture of mouse SMCs was established by explant outgrowth of a segment of human umbilical cord retrieved at the time of caesarean section [20]. Endothelial cells were removed by scraping the luminal surface of the vessel with a cotton swab, and the adventitia was mechanically stripped away. To induce calcification, VSMCs were maintained in DMEM with L-glutamine, sodium pyruvate, 10 mmol/L β-glycerophosphate, 10% FBS and 1% penicillin/streptomycin at 37°C, 5% CO_2_ incubator. We performed our further experimentations by using the cells at a density of 10^5^/well in six-well plates.

### SiRNA interference

siRNA method was performed to inhibit TXNIP, AMPKα and Atg 5 expression. Briefly, VSMCs were transfected with TXNIP, AMPKα or Atg 5 siRNA with Lipofectamine^®^ 2000 according to the manufacturer’s instructions. The process of transfection was performed in the absence of antibiotics. Following 24h incubation, the cells were used for other experiments. siRNA was synthesized by Biotend (Shanghai, China).

### Alizarin Red staining

The arterial samples were fixed with 4% formaldehyde and embedded in paraffin. Then, the paraffin-embedded tissues were cut into 4 μm-thick sections for subsequent analysis. After deparaffinization and rinsing in ethanol, sections were rinsed in distilled water for 5 minutes. The slices were dyed with 1% Alizarin Red S solution for 30 min and then photographed. VSMCs were fixed with 4% paraformaldehyde for 5 min at room temperature, washed three times with Ca^2+^-free PBS, and then stained with 1% Alizarin red solution (Beyotime, Shanghai, China) for 30 min at 37°C to visualize the matrix calcium deposition. The excess Alizarin red S reagent were washed out with Ca^2+^-free PBS and the samples were photographed.

### ROS determination

Intracellular ROS level was determined with the dihydroethidium (DHE) reagent (Sigma-Aldrich, USA). The treated cells were washed with PBS solution and incubated with 10 μM DHE for 30 min at 37°C in darkness. Then, VSMCs were washed twice using PBS and examined by fluorescence microscopy.

### Animals and *in vivo* experiments

The animal experiments were conducted on healthy male C57BL/6 mice weighing 20–25 g at 6 weeks of age, the mice were kept in a 12h dark/light cycle room with constant temperature (22–25°C). All experiments on mice were approved by the committee of Ruijing Hospital Academy of Medical Sciences. All the mice were randomly divided into four groups (*n* = 6 for each group): (I) control group; (II) galectin-3 treatment group (GAL); (III) galectin-3 and verapamil cotreatment group. Galectin-3 (20 μg) was intraperitoneally injected or the same volume of vehicle (saline) was conducted on every 3 days for a total of 4 weeks. Verapamil (100 mg/kg) was intraperitoneally injected every day. All mice were euthanized 4 weeks after the initial injection of galectin-3.

### Western blot

Cells were lysed with 100 mM phenylmethanesulfonyl fluoride. Protein concentrations were measured with the BCA Protein Assay. The lysates (20 μg) were electrophoresed on 10% SDS-PAGE and transferred to nitrocellulose membranes (Merck Millipore, Danvers, MA, USA). The membrane was blocked with 5% nonfat dry milk in TBST buffer (100 mM NaCl, 10 mM Tris-HCl, pH 7.4, and 0.1% Tween-20) for 1h at room temperature. The membrane was then incubated with diluted primary antibodies at 4°C overnight, and then washed twice with TBST buffer and incubated for 1 h with secondary antibody at room temperature. Image J was used to quantity the protein by assessing band intensity.

### Statistical analysis

Data were presented as the mean ± SEM and analyzed by one-way ANOVA followed by the Student-Newman–Keuls post hoc analyses when appropriate. Non-parametric ANOVA (Kruskal-Wallis test) was used, when the data were not passed the normality test. *P* < 0.05 was considered statistically significant. All experiments were performed at least three times.

### Data availability

The datasets used during the current study are available from the corresponding author on reasonable request.

## RESULTS

### Galectin-3 induced aorta and VSMCs calcification

**Figure 1:**
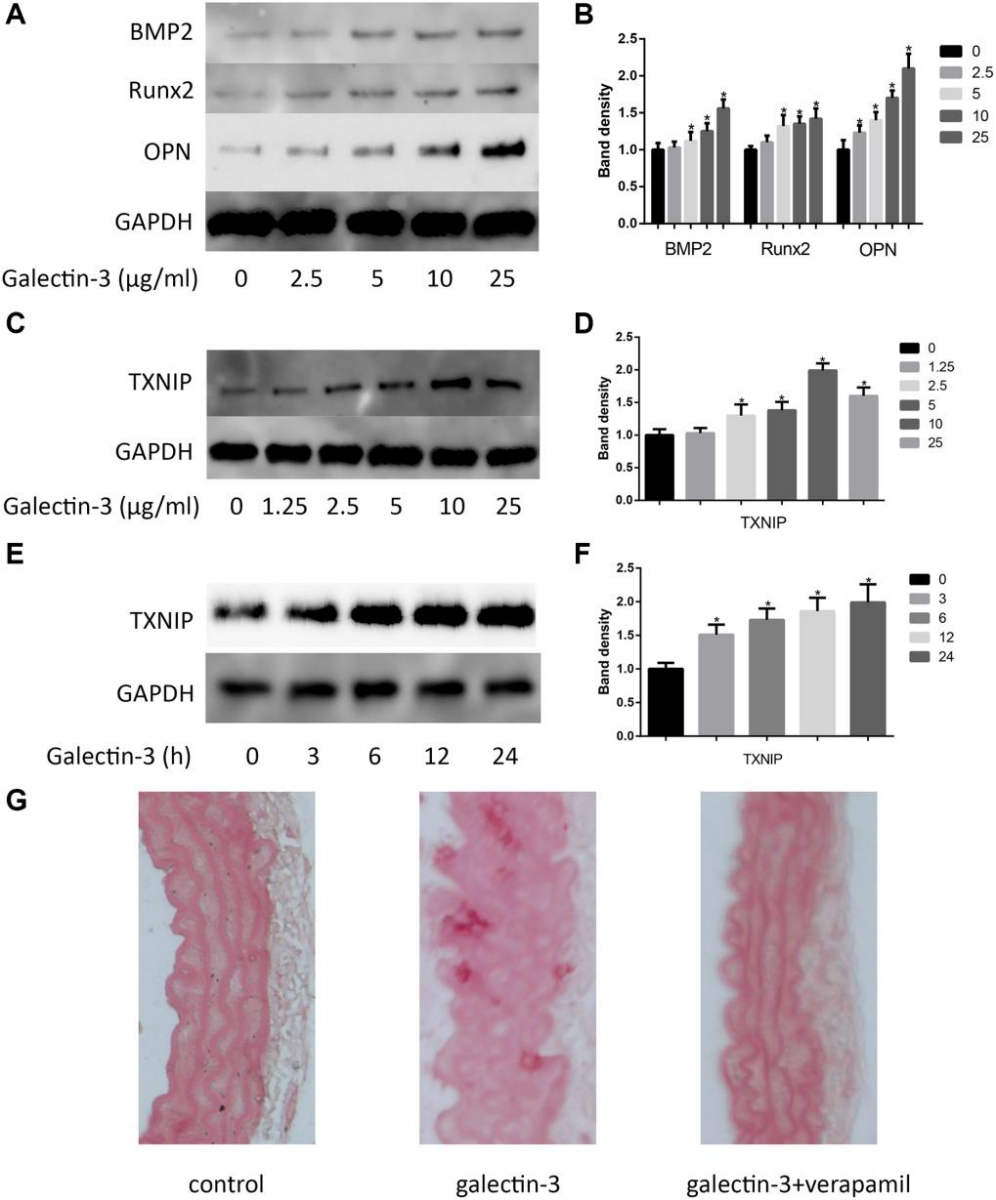
**Galectin-3 induced aorta and VSMCs calcification.** Cells were treated with galectin-3 over a range of concentrations (0, 2.5 μg/ml, 5 μg/ml, 10 μg/ml, 25 μg/ml) for 24 h, VSMCs osteogenic differentiation proteins (BMP2, Runx2 and OPN) were measured by Western blot, the quantification result is shown in the right panel (**A** and **B**). VSMCs were treated with galectin-3 over a range of concentrations (0, 1.25 μg/ml, 2.5 μg/ml, 5 μg/ml, 10 μg/ml, 25 μg/ml) for 24 h, TXNIP expression was measured by Western blot (**C**), the quantification result is shown in the right panel (**D**). 10 μg/ml galectin-3 was used to deal with VSMCs for different times (0, 3 h, 6 h, 12 h, 24 h), TXNIP expression was measured by Western blot (**E**), the quantification result is shown in the right panel (**F**). Band density of native VSMCs was defined as a control and considered to 1. Representative images of Alizarin Red staining of aorta (**G**). All experiments were performed at least three times. ^*^P < 0.05 compared with control.

In order to investigate the effects of galectin-3 on VSMCs calcification, VSMCs were cultured in DMEM without serum for 12 h, after that, the serum-starved VSMCs were treated with different concentration of galectin-3 for 24 h, we observed the expression of osteogenic markers, including BMP2, Runx2 and OPN. As shown in Figure 1A and 1B, the expression of BMP2, Runx2 and OPN was upregulated after galectin-3 treatment. As TXNIP, an important protein of inflammation, may also participate in vascular calcification [7], we observed TXNIP expression after the treatment of different concentration of galectin-3 (0, 1.25, 2.5, 5, 10 and 25 μg/ml), 10 μg/ml galectin-3 most effectively increased TXNIP expression. We then used 10 μg/ml galectin-3 to deal with VSMCs for different times, TXNIP was obviously upregulated by galectin-3 treatment for 3 h, and was increased significantly until 24 h galectin-3 treatment (Figure 1C–1F). Since TXNIP was also increased after galectin-3 treatment, we next determined the effect of TXNIP in galectin-3-induced aorta and VSMCs calcification. *In vivo*, we injected verapamil and (or) galectin-3 into mice and observed calcium deposition in aortic tissues via Alizarin Red Staining. There was no calcified deposition in aortas of control group, galectin-3 significantly increased positive staining among elastic fibers of aortal media. Verapamil, a TXNIP inhibitor, effectively decreased galectin-3-induced artery calcification (Figure 1G).

### TXNIP mediated galectin-3-induced VSMCs autophagy and osteogenic differentiation

**Figure 2:**
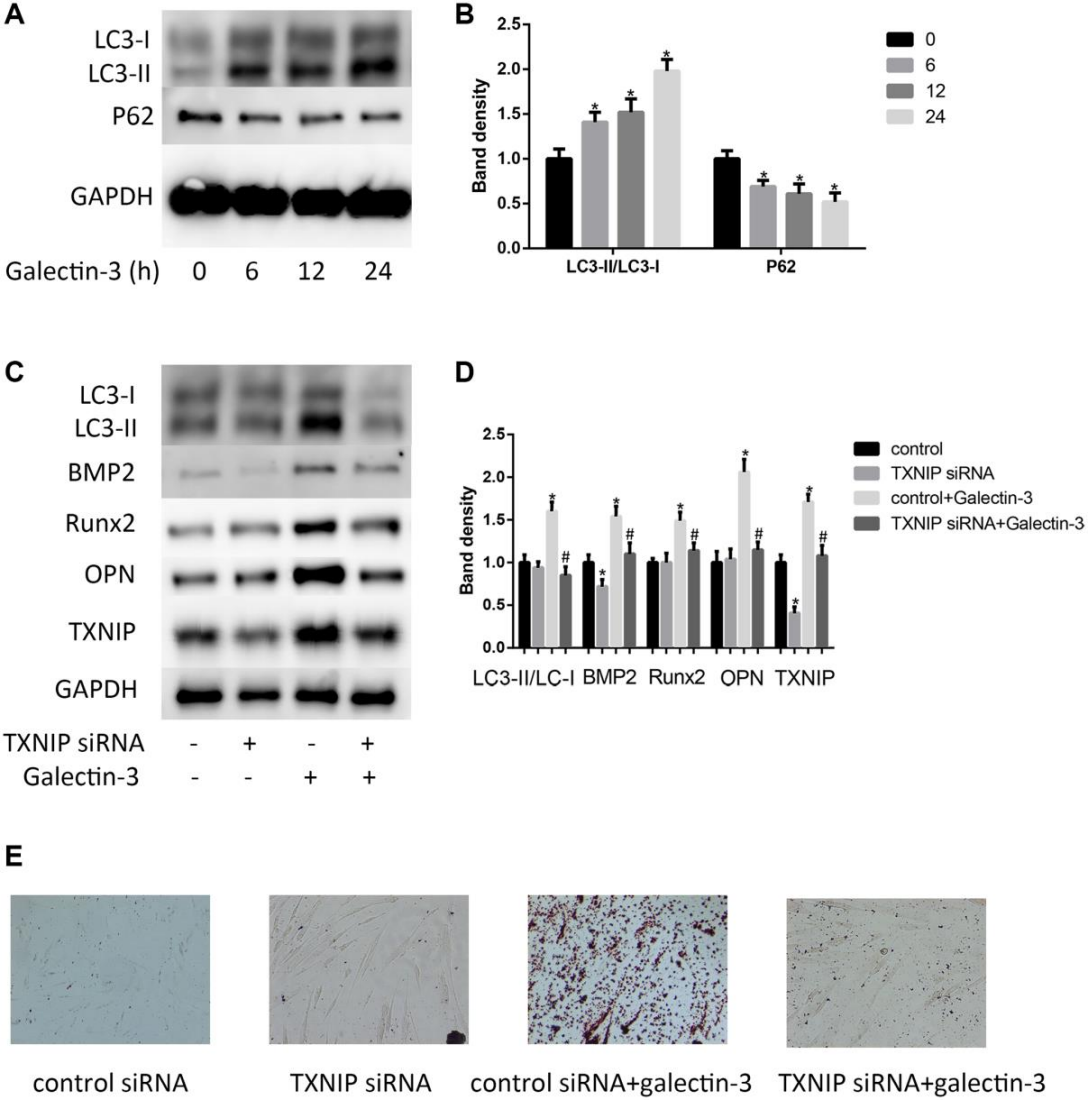
**Galectin-3 induced VSMCs calcification and autophagy via TXNIP.** VSMCs were treated with 10 μg/ml galectin-3 for different times (0, 6 h, 12 h, 24 h). The protein expression level of LC3-II/LC3-I and P62 were measured by western blot, the results quantifications were shown in the right panel (**A** and **B**). After transfection with either control or TXNIP siRNA for 24 h, VSMCs were incubated in the absence or presence of 10 μg/ml galectin-3 for 24 h, the expression of LC3-II/LC3-I and VSMCs osteogenic differentiation proteins (BMP2, Runx2 and OPN) was measured by western blot, the results quantifications were shown in the right panel (**C** and **D**). Band density of native VSMCs was defined as a control and considered to 1. After transfection with either control or TXNIP siRNA for 24 h, VSMCs were incubated in the absence or presence of 10μg/ml galectin-3 for 7 d, Alizarin red staining was used to observe the calcium deposition (**E**). Data were obtained from three independent experiments. ^*^P < 0.05 vs. control; ^#^*P* < 0.05 vs. galectin-3.

In atherosclerotic plaque, there is an increase expression of autophagy related proteins [21]. In order to explore the possible mechanisms of how TXNIP mediated galectin-3-induced VSMCs osteogenic differentiation, we focus on the autophagy process. We found that after galectin-3 treatment for 24 h, there was a time-dependent increase in LC3-II formation and decrease in p62 expression. A significant increase in LC3-II was observed 6 h after galectin-3 treatment, in contrast, a selective substrate of the autophagy degrading pathway p62 was decreased after galectin-3 treatment (Figure 2A and 2B). We furtherly verified TXNIP effect on autophagy, TXNIP siRNA decreased galectin-3-induced upregulation of LC3-II/LC3-I. Our results suggest that TXNIP mediates galectin-3-induced autophagy in VSMCs.

We then verified the role of TXNIP in galectin-3-induced VSMCs calcification. We examined BMP2, Runx2 and OPN expression in VSMCs after TXNIP siRNA treatment. As shown in Figure 2C and 2D, TXNIP knockdown has little effect on BMP2, Runx2 and OPN expression in basal condition, but it significantly attenuated galectin-3-induced upregulation of these proteins. These results suggested that TXNIP mediated galectin-3-induced expression of VSMCs osteogenic differentiation. Alizarin red staining was used to observe the calcium deposition. Alizarin red staining showed that galectin-3 significantly increased calcium content in VSMCs and that TXNIP siRNA blocked calcium deposition in cells (Figure 2E).

### TXNIP knockdown decreases galectin-3-induced ROS and inflammation factors expression in VSMCs

**Figure 3:**
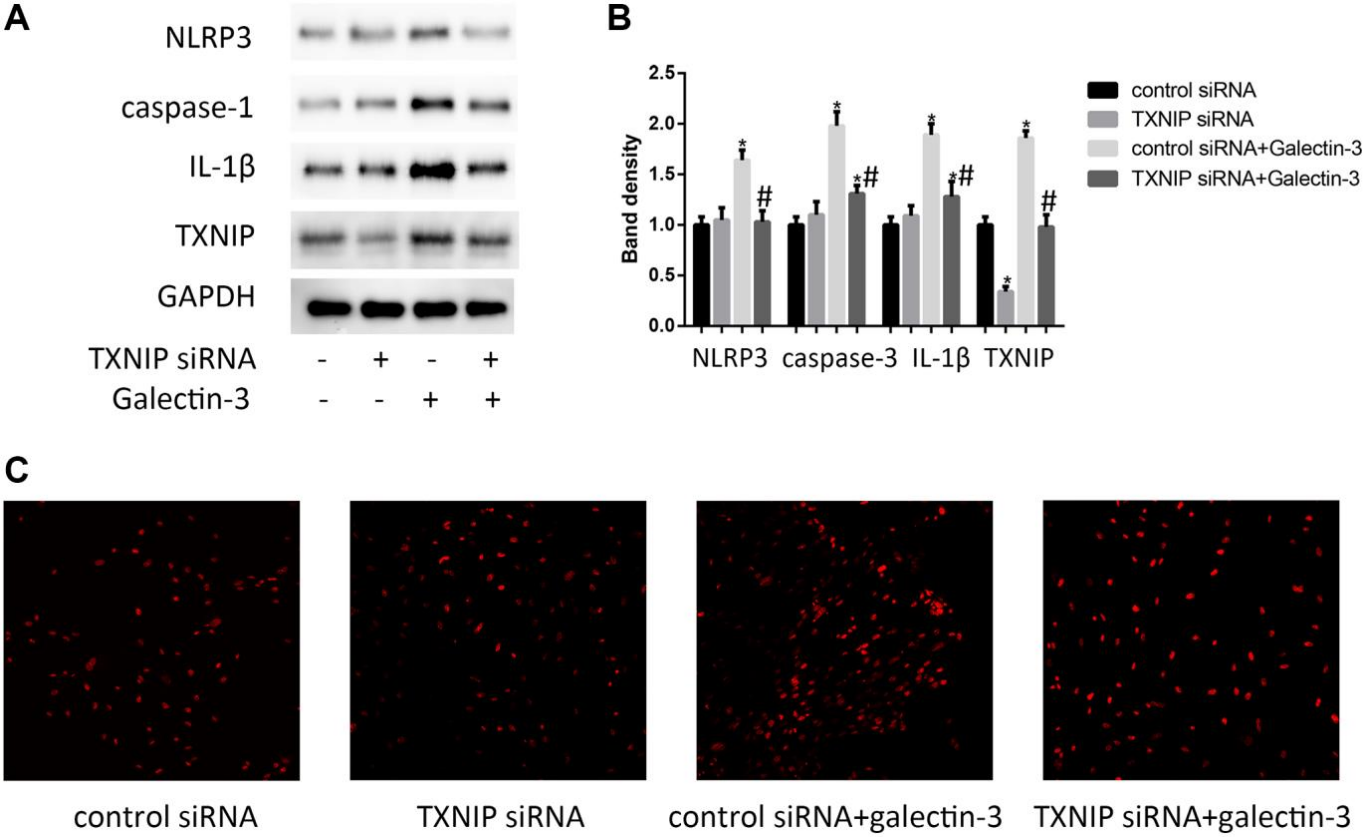
**TXNIP mediated galectin-3-induced ROS and inflammation factors expression in VSMCs.** VSMCs were treated with 10 μg/ml galectin-3 and (or) TXNIP siRNA for 24 h, The protein expression level of NLRP3, caspase-1, and IL-1β were measured by western blot, the results quantifications were shown in the right panel (**A** and **B**). Band density of native VSMCs was defined as a control and considered to 1. DHE staining was used to observe the ROS production (**C**). Data were obtained from three independent experiments. ^*^P < 0.05, vs. the control; ^#^*P* < 0.05 vs. galectin-3.

TXNIP binding to NLRP3 inflammasome is involved in regulating inflammation [14], besides, inflammation plays a key role in vascular calcification. In order to verify TXNIP effect on the expression of NLRP3 inflammasome (NLRP3 and caspase-1) and IL-1β in galectin-3 treatment VSMCs, the protein and mRNA level of NLRP3, caspase-1, and IL-1β were observed by using Western blot and qRT-PCR respectively. As shown in Figure 3A and 3B, galectin-3 significantly increased NLRP3, caspase-1, and IL-1β expression. The VSMCs with verapamil and galectin-3 treatment showed less expression of NLRP3, caspase-1, and IL-1β compared with galectin-3 treated VSMCs. The result was similar in Figure 3C, which showed increased ROS production after galectin-3 treatment was reversed by TXNIP siRNA treatment in VSMCs.

### Autophagy mediated galectin-3-induced VSMCs osteogenic differentiation

**Figure 4:**
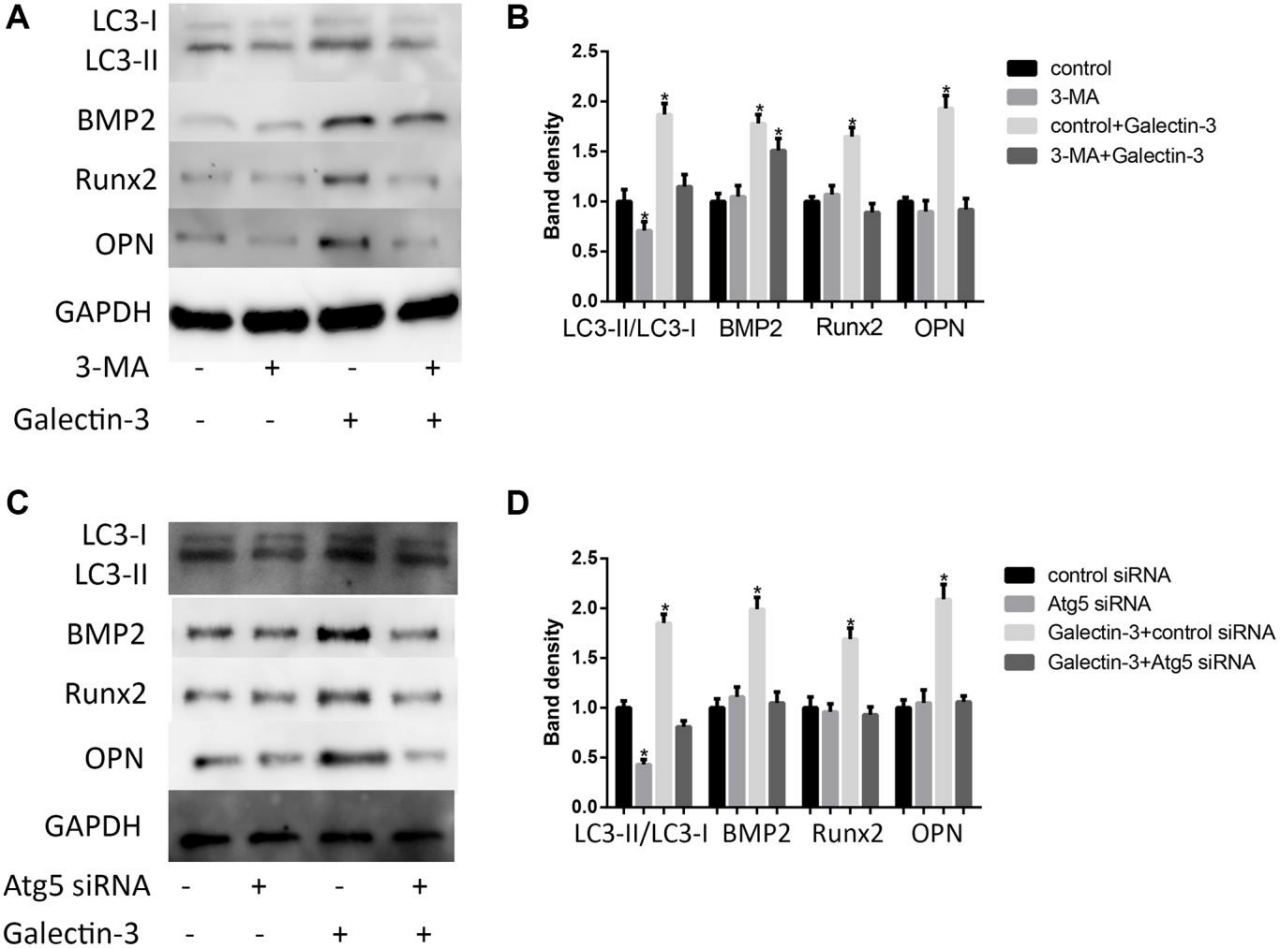
**Autophagy mediated galectin-3-induced VSMCs calcification.** After pre-treatment with 100 mmol/L 3-MA for 1 h, the VSMCs were then treated with 10 μg/ml galectin-3 for 24 h, BMP2, Runx2 and OPN expression were measured by Western blot, the results quantifications were shown in the right panel (**A** and **B**). After treatment with Atg5 siRNA for 24 h, the VSMCs were then treated with 10 μg/ml galectin-3 for 24 h, BMP2, Runx2 and OPN expression were measured by Western blot, the results quantifications were shown in the right panel (**C** and **D**). Band density of native VSMCs was chosen as a reference and set to 1. Data were obtained from three independent experiments. ^*^P < 0.05, vs. the control.

Until now, the role of autophagy in VSMCs osteogenic differentiation is still unclear, we would like to explore the relationship between autophagy and VSMCs osteogenic differentiation. We hypothesized that autophagy might mediate galectin-3-induced VSMCs differentiation. To test this hypothesis, we used autophagy inhibitors, 3-MA (100 mmol/L) or Atg5 siRNA, to treat VSMCs before galectin-3 stimulation. 3-MA or Atg5 siRNA not only effectively decreased the LC3-II protein expression (Figure 4A and 4B) but also inhibited galectin-3-induced upregulation of BMP2, Runx2 and OPN expression (Figure 4C and 4D).

### AMPK signaling pathway mediated galectin-3-induced TXNIP expression and VSMCs osteogenic differentiation

**Figure 5:**
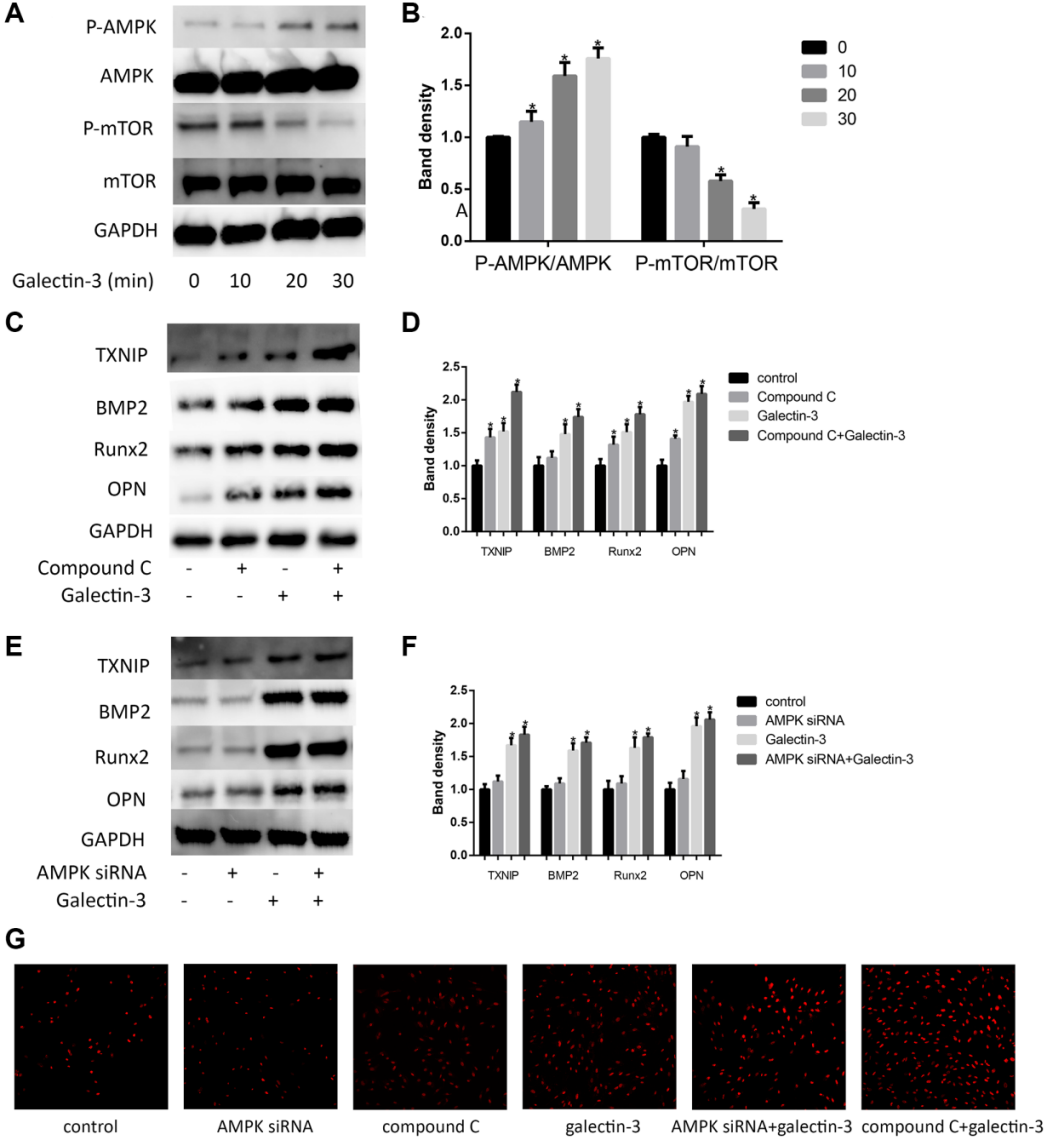
**AMPK signaling pathway mediated galectin-3-induced TXNIP and VSMCs osteogenic differentiation.** Cells were treated with 10 μg/ml galectin-3 over a range of times (0–60 min), and the expression of AMPK, p-AMPK, mTOR, and p-mTOR were measured by Western blot. Western blot results are shown, quantification of the results is given in the right panel (**A** and **B**). Band density of native VSMCs was defined as a control and considered to 1. Data were obtained from three independent experiments. ^*^P < 0.05 compared with control. After pre-treatment with 1 μmol/L compound C for 1 h, VSMCs were then treated with 10 μg/ml galectin-3 for 24 h, the expression of TXNIP and VSMCs osteogenic differentiation proteins was measured by western blot (**C**). Quantification of the results were shown in the right panel (**D**). After pre-treatment with AMPK siRNA for 24 h, VSMCs were then treated with 10 μg/ml galectin-3 for 24 h, the expression of TXNIP and VSMCs osteogenic differentiation proteins was measured by western blot (**E**). Quantification of the results were shown in the right panel (**F**). Band density of native VSMCs was defined as a control and considered to 1. DHE staining was used to observe the ROS production (**G**). Data were obtained from three independent experiments. ^*^P < 0.05 vs. control.

AMPK has also been regarded as a mTOR-independent manner to induce the up-regulation of autophagic activity, besides, phosphorylation of AMPK has also been shown to mediate VSMCs differentiation [22]. Here, we examined mTOR and AMPK signaling pathways after galectin-3 treatment. We used 10 μg/ml galectin-3 to deal with VSMCs for 60 min, p-mTOR was effectively suppressed after galectin-3 treatment. These results furtherly confirmed that galectin-3 activated autophagy process in VSMCs (Figure 5A and 5B). We also observed AMPK signaling pathway after galectin-3 treatment, galectin-3 significantly increased p-AMPK expression, and there was no difference of total AMPK level after galectin-3 treatment (Figure 5A and 5B).

We then verified the role of AMPK signaling pathway in galectin-3-induced TXNIP expression and VSMCs osteogenic differentiation. we pretreated VSMCs with a specific AMPK inhibitor compound C (1 μmol/L) or AMPKα specific siRNA, and then examined TXNIP expression. Either compound C or AMPKα specific siRNA significantly enhanced galectin-3-induced upregulation of TXNIP (Figure 5C–5F). To furtherly explore whether galectin-3 induced VSMCs osteogenic differentiation via AMPK, we also pretreated VSMCs with compound C or AMPKα siRNA before galectin-3 treatment. As shown in Figure 5C and 5E, AMPK signal blockade furtherly increased the expression of OPN, Runx2 and BMP2 in galectin-3-treated VSMCs, besides, we also observed ROS production via DHE staining, AMPK inhibition can also promote galectin-3-induced ROS expression (Figure 5G).

## DISCUSSION

Galectin-3 plays an important role in vascular calcification. In unstable plaque regions, galectin-3 was found to be expressed by inflammatory cells, moreover, it was upregulated by VSMCs in sheet-like/lamellated calcification areas [23]. In our previous article, galectin-3 was proved to mediate oxLDL-induced VSMCs phenotype transformation [24]. TXNIP has been regarded as a mediator in activation of the reactive oxygen species (ROS)-induced inflammasome [25]. However, until now the relationship between galectin-3 and TXNIP and its regulation effects on vascular calcification is still unclear. Here, we found that TXNIP was essential for galectin-3-induced VSMCs calcification, in addition, AMPK signaling pathway was involved in this process.

Autophagy is an important protective mechanism for cell survival [26]. We have also found that galectin-3 increased the VSMCs proliferation and migration [27]. An increase autophagy has been observed in vascular calcification [19]. Autophagy involves many important molecules, such as LC3B (autophagosome membrane expansion and fusion) and p62 (cargo receptors involved in selective autophagy) [28]. In VSMCs, we found that galectin-3 induced autophagy by increasing LC3-II/ LC3-I expression, as well as decreasing p62 expression. AMPK signaling pathway has been proved to regulate autophagy processes [29, 30]. In our article, we found that galectin-3 could induce autophagy and activate AMPK signaling pathways. Galectin-3 induces VSMCs autophagy, possibly through the AMPK signaling pathways, which may play a protective role against galectin-3-induced VSMCs calcification.

AMPK signaling pathways play an important role in TXNIP expression. MPK pathway is essential for cell survival [31]. AMPK pathway is also largely involved in catabolism, mitochondrial activation, and increases cell viability [32]. This is because AMPK, an energy-sensitive serine/threonine protein kinase, is required to maintain intracellular NADPH levels in response to metabolic stress and energy deprivation [33, 34]. We thus determined to test whether galectin-3 induced TXNIP expression via AMPK. In our researches, after specific blocking AMPK, galectin-3 could furtherly increase TXNIP expression. It is well-known that AMPK activation leads to TXNIP degeneration. However, in galectin-3 treatment VSMCs, TXNIP expression is significantly upregulated, it seems that AMPK activation is not sufficient for TXNIP degeneration, TXNIP desensitization may attenuate efficiency of P-AMPK upregulation on TXNIP degeneration [35], besides the AMPK activation and TXNIP degeneration are also observed in trophoblasts during the development of preeclampsia and hyperglycemia-induced cardiomyocytes [35, 36].

TXNIP is the cell surface receptor which has been implicated in vascular inflammation. Oxidative inflammatory response can induce atherosclerotic lesion development and progression [37], however, the relationship between TXNIP and vascular calcification has not been previously elucidated. In podocytes, TXNIP deficiency mitigate the mTOR signaling activation, apoptosis-related proteins and HG (high glucose)-induced apoptosis [38]. Byon et al. found that TXNIP knockout mice have decrease expression of inflammatory markers and adhesion molecules in VSMCs and protect from oxidative stress after treatment with oxidized phospholipids and hydrogen peroxide [7]. We discovered that TXNIP siRNA attenuated galectin-3-induced VSMCs osteogenic differentiation, inflammatory responses and ROS production. Our results indicate that galectin-3 accelerates VSMCs calcification at least partly through a TXNIP-dependent pathway. The atheroprotective effect of TXNIP ablation implicates that modulation of TXNIP expression may serve as a potential target for intervention of atherosclerosis and inflammatory vascular disease.

Taken together, our study demonstrated that galectin-3 induced VSMCs calcification via TXNIP and autophagy in VSMCs, which was significantly mediated by modulating AMPK signaling pathways. As smooth muscle cells and galectin-3 play an important role in a variety of plausible mechanisms of atherosclerosis and chronic heart diseases. Thus, our work could help to clarify the role of TXNIP in the pathological process of atherosclerosis and open up a new insight into the regulatory mechanism of VSMCs calcification in combating plaque rupture by galectin-3.

## References

[1] Cimmino G, Ragni M, Cirillo P, Petrillo G, Loffredo F, Chiariello M, Gresele P, Falcinelli E, Golino P. C-reactive protein induces expression of matrix metalloproteinase-9: a possible link between inflammation and plaque rupture. Int J Cardiol. 2013; 168:981–6. 10.1016/j.ijcard.2012.10.040 23157807

[2] Newby AC. Metalloproteinase expression in monocytes and macrophages and its relationship to atherosclerotic plaque instability. Arterioscler Thromb Vasc Biol. 2008; 28:2108–14. 10.1161/ATVBAHA.108.173898 18772495

[3] Yoon YW, Kwon HM, Hwang KC, Choi EY, Hong BK, Kim D, Kim HS, Cho SH, Song KS, Sangiorgi G. Upstream regulation of matrix metalloproteinase by EMMPRIN; extracellular matrix metalloproteinase inducer in advanced atherosclerotic plaque. Atherosclerosis. 2005; 180:37–44. 10.1016/j.atherosclerosis.2004.11.021 15823273

[4] Ozturk D, Celik O, Satilmis S, Aslan S, Erturk M, Cakmak HA, Kalkan AK, Ozyilmaz S, Diker V, Gul M. Association between serum galectin-3 levels and coronary atherosclerosis and plaque burden/structure in patients with type 2 diabetes mellitus. Coron Artery Dis. 2015; 26:396–401. 10.1097/MCA.0000000000000252 25887000

[5] Wang Z, Chen Z, Ma X, Yu H, Chen X. The predictive value of serum galectin 3 for abdominal aortic calcification in maintenance hemodialysis patients: A prospective cohort study. Hemodial Int. 2020; 24:212–20. 10.1111/hdi.12825 32048459

[6] Pugliese G, Iacobini C, Blasetti Fantauzzi C, Menini S. The dark and bright side of atherosclerotic calcification. Atherosclerosis. 2015; 238:220–30. 10.1016/j.atherosclerosis.2014.12.011 25528431

[7] Byon CH, Han T, Wu J, Hui ST. Txnip ablation reduces vascular smooth muscle cell inflammation and ameliorates atherosclerosis in apolipoprotein E knockout mice. Atherosclerosis. 2015; 241:313–21. 10.1016/j.atherosclerosis.2015.05.020 26062991 PMC4509824

[8] Andres AM, Ratliff EP, Sachithanantham S, Hui ST. Diminished AMPK signaling response to fasting in thioredoxin-interacting protein knockout mice. FEBS Lett. 2011; 585:1223–30. 10.1016/j.febslet.2011.03.042 21439280 PMC3088363

[9] Chutkow WA, Patwari P, Yoshioka J, Lee RT. Thioredoxin-interacting protein (Txnip) is a critical regulator of hepatic glucose production. J Biol Chem. 2008; 283:2397–406. 10.1074/jbc.M708169200 17998203

[10] Yoshihara E, Masaki S, Matsuo Y, Chen Z, Tian H, Yodoi J. Thioredoxin/Txnip: redoxisome, as a redox switch for the pathogenesis of diseases. Front Immunol. 2014; 4:514. 10.3389/fimmu.2013.00514 24409188 PMC3885921

[11] Hwang J, Suh HW, Jeon YH, Hwang E, Nguyen LT, Yeom J, Lee SG, Lee C, Kim KJ, Kang BS, Jeong JO, Oh TK, Choi I, et al. The structural basis for the negative regulation of thioredoxin by thioredoxin-interacting protein. Nat Commun. 2014; 5:2958. 10.1038/ncomms3958 24389582 PMC3941024

[12] Ferreira NE, Omae S, Pereira A, Rodrigues MV, Miyakawa AA, Campos LC, Santos PC, Dallan LA, Martinez TL, Santos RD, Mill JG, Krieger JE, Pereira AC. Thioredoxin interacting protein genetic variation is associated with diabetes and hypertension in the Brazilian general population. Atherosclerosis. 2012; 221:131–6. 10.1016/j.atherosclerosis.2011.12.009 22236479

[13] Alvim RO, Santos PC, Ferreira NE, Mill JG, Krieger JE, Pereira AC. Thioredoxin interacting protein (TXNIP) rs7212 polymorphism is associated with arterial stiffness in the Brazilian general population. J Hum Hypertens. 2012; 26:340–2. 10.1038/jhh.2011.102 22113441

[14] Jiang J, Shi Y, Cao J, Lu Y, Sun G, Yang J. Role of ASM/Cer/TXNIP signaling module in the NLRP3 inflammasome activation. Lipids Health Dis. 2021; 20:19. 10.1186/s12944-021-01446-4 33612104 PMC7897379

[15] Wang Y, Ma WQ, Zhu Y, Han XQ, Liu N. Exosomes Derived From Mesenchymal Stromal Cells Pretreated With Advanced Glycation End Product-Bovine Serum Albumin Inhibit Calcification of Vascular Smooth Muscle Cells. Front Endocrinol (Lausanne). 2018; 9:524. 10.3389/fendo.2018.00524 30298051 PMC6160580

[16] Zhao J, Zheng H, Sui Z, Jing F, Quan X, Zhao W, Liu G. Ursolic acid exhibits anti-inflammatory effects through blocking TLR4-MyD88 pathway mediated by autophagy. Cytokine. 2019; 123:154726. 10.1016/j.cyto.2019.05.013 31302461

[17] Chen J, Gu Y, Zhang H, Ning Y, Song N, Hu J, Cai J, Shi Y, Ding X, Zhang X. Amelioration of Uremic Toxin Indoxyl Sulfate-Induced Osteoblastic Calcification by SET Domain Containing Lysine Methyltransferase 7/9 Protein. Nephron. 2019; 141:287–94. 10.1159/000495885 30783062

[18] Sun Y, Byon CH, Yang Y, Bradley WE, Dell'Italia LJ, Sanders PW, Agarwal A, Wu H, Chen Y. Dietary potassium regulates vascular calcification and arterial stiffness. JCI Insight. 2017; 2:94920. 10.1172/jci.insight.94920 28978809 PMC5841863

[19] Xu M, Liu L, Song C, Chen W, Gui S. Ghrelin improves vascular autophagy in rats with vascular calcification. Life Sci. 2017; 179:23–9. 10.1016/j.lfs.2016.11.025 27916732

[20] Guyton JR, Lenz ML, Mathews B, Hughes H, Karsan D, Selinger E, Smith CV. Toxicity of oxidized low density lipoproteins for vascular smooth muscle cells and partial protection by antioxidants. Atherosclerosis. 1995; 118:237–49. 10.1016/0021-9150(95)05610-6 8770318

[21] Zhou X, Xu SN, Yuan ST, Lei X, Sun X, Xing L, Li HJ, He CX, Qin W, Zhao D, Li PQ, Moharomd E, Xu X, Cao HL. Multiple functions of autophagy in vascular calcification. Cell Biosci. 2021; 11:159. 10.1186/s13578-021-00639-9 34399835 PMC8369777

[22] Thompson AM, Martin KA, Rzucidlo EM. Resveratrol induces vascular smooth muscle cell differentiation through stimulation of SirT1 and AMPK. PLoS One. 2014; 9:e85495. 10.1371/journal.pone.0085495 24416418 PMC3885718

[23] Menini S, Iacobini C, Ricci C, Blasetti Fantauzzi C, Salvi L, Pesce CM, Relucenti M, Familiari G, Taurino M, Pugliese G. The galectin-3/RAGE dyad modulates vascular osteogenesis in atherosclerosis. Cardiovasc Res. 2013; 100:472–80. 10.1093/cvr/cvt206 23975852

[24] Tian L, Chen K, Cao J, Han Z, Gao L, Wang Y, Fan Y, Wang C. Galectin-3-induced oxidized low-density lipoprotein promotes the phenotypic transformation of vascular smooth muscle cells. Mol Med Rep. 2015; 12:4995–5002. 10.3892/mmr.2015.4075 26165519 PMC4581830

[25] Martinon F, Mayor A, Tschopp J. The inflammasomes: guardians of the body. Annu Rev Immunol. 2009; 27:229–65. 10.1146/annurev.immunol.021908.132715 19302040

[26] Grootaert MOJ, Moulis M, Roth L, Martinet W, Vindis C, Bennett MR, De Meyer GRY. Vascular smooth muscle cell death, autophagy and senescence in atherosclerosis. Cardiovasc Res. 2018; 114:622–34. 10.1093/cvr/cvy007 29360955

[27] Tian L, Chen K, Cao J, Han Z, Wang Y, Gao L, Fan Y, Wang C. Galectin-3 induces the phenotype transformation of human vascular smooth muscle cells via the canonical Wnt signaling. Mol Med Rep. 2017; 15:3840–6. 10.3892/mmr.2017.6429 28393190

[28] Vishnupriya S, Priya Dharshini LC, Sakthivel KM, Rasmi RR. Autophagy markers as mediators of lung injury-implication for therapeutic intervention. Life Sci. 2020; 260:118308. 10.1016/j.lfs.2020.118308 32828942 PMC7442051

[29] Chiou JT, Huang CH, Lee YC, Wang LJ, Shi YJ, Chen YJ, Chang LS. Compound C induces autophagy and apoptosis in parental and hydroquinone-selected malignant leukemia cells through the ROS/p38 MAPK/AMPK/TET2/FOXP3 axis. Cell Biol Toxicol. 2020; 36:315–31. 10.1007/s10565-019-09495-3 31900833

[30] Zhao X, Luo G, Cheng Y, Yu W, Chen R, Xiao B, Xiang Y, Feng C, Fu W, Duan C, Yao F, Xia X, Tao Q, et al. Compound C induces protective autophagy in human cholangiocarcinoma cells via Akt/mTOR-independent pathway. J Cell Biochem. 2018; 119:5538–50. 10.1002/jcb.26723 29384220

[31] Jeon SM, Chandel NS, Hay N. AMPK regulates NADPH homeostasis to promote tumour cell survival during energy stress. Nature. 2012; 485:661–5. 10.1038/nature11066 22660331 PMC3607316

[32] Carling D. AMPK signalling in health and disease. Curr Opin Cell Biol. 2017; 45:31–7. 10.1016/j.ceb.2017.01.005 28232179

[33] Wu KC, Cui JY, Klaassen CD. Beneficial role of Nrf2 in regulating NADPH generation and consumption. Toxicol Sci. 2011; 123:590–600. 10.1093/toxsci/kfr183 21775727 PMC3179677

[34] Li K, Liu TX, Li JF, Ma YR, Liu ML, Wang YQ, Wu R, Li B, Shi LZ, Chen C. rhEPO inhibited cell apoptosis to alleviate acute kidney injury in sepsis by AMPK/SIRT1 activated autophagy. Biochem Biophys Res Commun. 2019; 517:557–65. 10.1016/j.bbrc.2019.07.027 31383361

[35] Wei H, Bu R, Yang Q, Jia J, Li T, Wang Q, Chen Y. Exendin-4 Protects against Hyperglycemia-Induced Cardiomyocyte Pyroptosis via the AMPK-TXNIP Pathway. J Diabetes Res. 2019; 2019:8905917. 10.1155/2019/8905917 31886288 PMC6925927

[36] Quan XZ, Ye JH, Yang XZ, Xie Y. HOXA9-induced chemerin signals through CMKLR1/AMPK/TXNIP/NLRP3 pathway to induce pyroptosis of trophoblasts and aggravate preeclampsia. Exp Cell Res. 2021; 408:112802. 10.1016/j.yexcr.2021.112802 34461109

[37] Grebe A, Hoss F, Latz E. NLRP3 Inflammasome and the IL-1 Pathway in Atherosclerosis. Circ Res. 2018; 122:1722–40. 10.1161/CIRCRESAHA.118.311362 29880500

[38] Song S, Qiu D, Wang Y, Wei J, Wu H, Wu M, Wang S, Zhou X, Shi Y, Duan H. TXNIP deficiency mitigates podocyte apoptosis via restraining the activation of mTOR or p38 MAPK signaling in diabetic nephropathy. Exp Cell Res. 2020; 388:111862. 10.1016/j.yexcr.2020.111862 31982382

